# Domestic cat nose functions as a highly efficient coiled parallel gas chromatograph

**DOI:** 10.1371/journal.pcbi.1011119

**Published:** 2023-06-29

**Authors:** Zhenxing Wu, Jianbo Jiang, Fritz W. Lischka, Scott J. McGrane, Yael Porat-Mesenco, Kai Zhao

**Affiliations:** 1 Department of Otolaryngology—Head & Neck Surgery, The Ohio State University, Columbus, Ohio, United States of America; 2 Monell Chemical Senses Center, Philadelphia, Pennsylvania, United States of America; 3 Waltham Petcare Science Institute, Freeby Lane, Waltham-on-the-Wolds, Melton Mowbray, Leicestershire, United Kingdom; 4 MJ Ryan Veterinary Hospital of the University of Pennsylvania, Philadelphia, Pennsylvania, United States of America; Université Paris Descartes, Centre National de la Recherche Scientifique, FRANCE

## Abstract

The peripheral structures of mammalian sensory organs often serve to support their functionality, such as alignment of hair cells to the mechanical properties of the inner ear. Here, we examined the structure-function relationship for mammalian olfaction by creating an anatomically accurate computational nasal model for the domestic cat (*Felis catus*) based on high resolution microCT and sequential histological sections. Our results showed a distinct separation of respiratory and olfactory flow regimes, featuring a high-speed dorsal medial stream that increases odor delivery speed and efficiency to the ethmoid olfactory region without compromising the filtration and conditioning purpose of the nose. These results corroborated previous findings in other mammalian species, which implicates a common theme to deal with the physical size limitation of the head that confines the nasal airway from increasing in length infinitely as a straight tube. We thus hypothesized that these ethmoid olfactory channels function as parallel coiled chromatograph channels, and further showed that the theoretical plate number, a widely-used indicator of gas chromatograph efficiency, is more than 100 times higher in the cat nose than an “amphibian-like” straight channel fitting the similar skull space, at restful breathing state. The parallel feature also reduces airflow speed within each coil, which is critical to achieve the high plate number, while feeding collectively from the high-speed dorsal medial stream so that total odor sampling speed is not sacrificed. The occurrence of ethmoid turbinates is an important step in the evolution of mammalian species that correlates to their expansive olfactory function and brain development. Our findings reveal novel mechanisms on how such structure may facilitate better olfactory performance, furthering our understanding of the successful adaptation of mammalian species, including *F*. *catus*, a popular pet, to diverse environments.

## Author summary

The evolutionary occurrence of convoluted ethmoid turbinate structures in mammalian nose, remarkably resembles a different sensory organ, the snail-like coiled cochlea that is also unique to mammals. In birds and other non-mammalian vertebrates, the inner hearing organ, despite being called “cochlea”, is instead a blind-ended tube. While the evolution of mammalian cochlea enhances our auditory frequency sensitivity and range, the functional benefit of the mammalian olfactory turbinates has only been speculated. Using anatomically-accurate computational models, we revealed that convoluted turbinate structures in the domestic cat, as a model, may function as a parallel coiled chromatograph to significantly increase odor delivery speed and chromatography efficiency: > 100 times higher, compared to an “amphibian-like” straight nasal channel fitting the same skull space.

## Introduction

The structures of mammalian sensory organs often serve to enhance their functionality based on incoming stimuli and the environment. For example, in vision, photoreceptors in plains-dwelling mammals (e.g., the rabbit) tend to concentrate into a horizontal band along the nasotemporal axis of the retina (i.e., the visual streak) [1,2], matching the horizon. Photoreceptors of other non-plains-dwelling species tend to concentrate in the center of the visual field (e.g., the fovea in primates). Nocturnal animals (e.g., cats) often have a reflective membrane (tapetum lucidum) immediately behind the retina to reflect light and increase sensitivity under dim lighting [3], which enables them to hunt at dawn and dusk. In hearing, the mechanical properties of the inner ear process sound waves in frequency domains as they propagate through the cochlea, to which inner hair cells with different frequency sensitivity are aligned [4]. Such structure-function relationships have enabled highly derived behavioral advantages in mammalian senses, such as echo location. The structure-function relationship is less known for the mammalian nose and the sense of smell. The olfactory receptors do not have direct access to stimuli—they are covered in a mucus layer. Thus, odorants must be absorbed into the mucus as they are inhaled with airflow into the nose depending on their physiochemical properties, prior to binding to the olfactory receptors, to which Mozell hypothesized that the nose may function as a gas chromatograph [5–7]. However, these earlier studies were mostly conducted in amphibians, with nasal structures much simpler to that of mammals. There is indirect evidence that the evolutionary occurrence of complex ethmoid turbinates as an important aspect of the complex structure in mammalian nose correlates with their improved olfaction performance [8] and brain development [9]. Previous computational studies have suggested that these ethmoid turbinates may contribute to directing different flow paths, regulate the amounts of bulk flow to/from olfactory regions, and increase levels of recirculation [10–13]. More recently, 3D transcriptomics has been used to examine the receptor gene distributions on the ethmoid turbinates in relationship to gas chromatograph theory [14], but there is still a need to address quantitatively what benefit is provided in terms of airflow and odorant absorption by the complex ethmoid structure of the mammalian nose.

**Fig 1:**
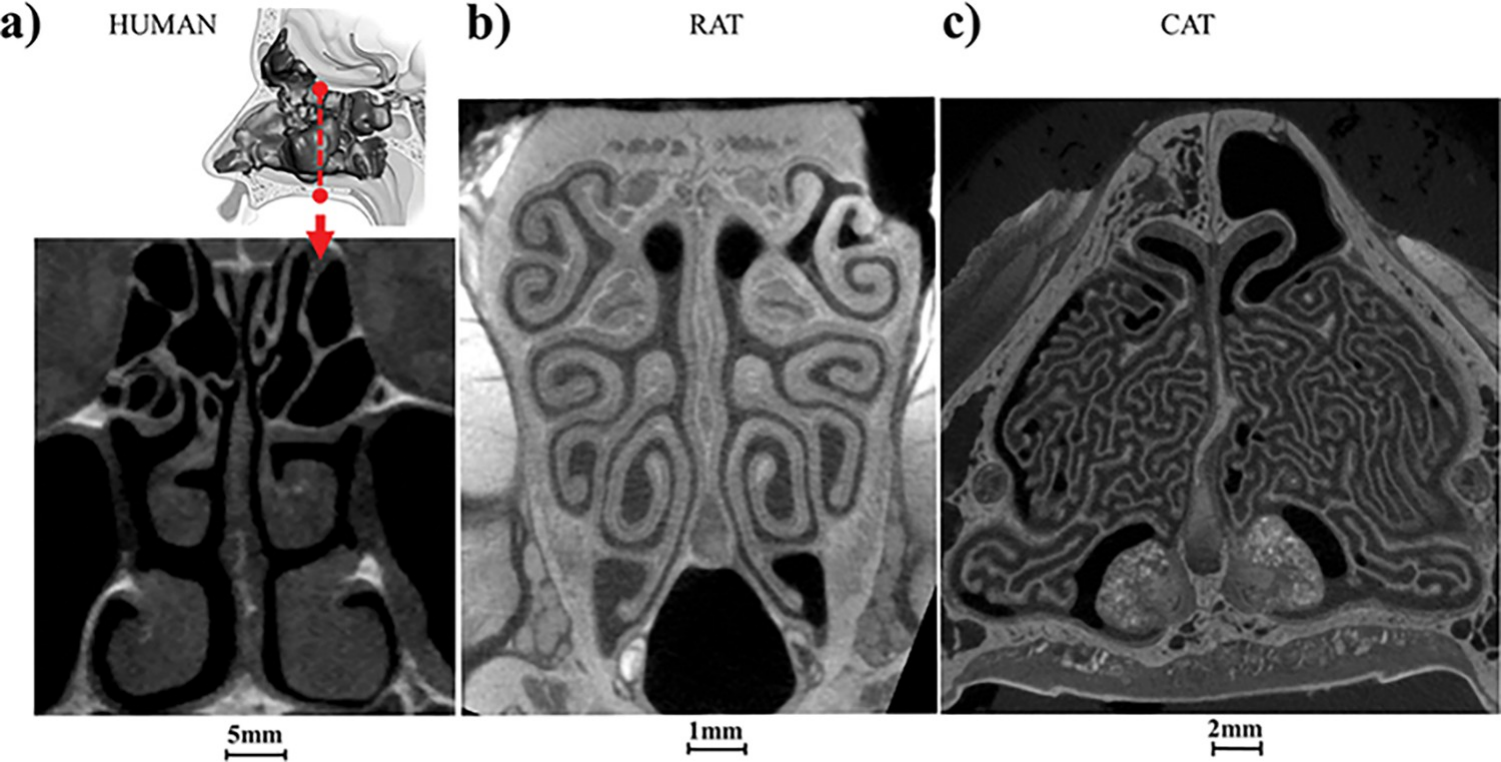
CT scans showing nasal cavity cross sections of a) human, b) rat, and c) domestic cat. The dark spaces on the scans are the nasal airways. As odorous air is drawn into the nose, it flows through these labyrinthine structures. The full set of CT scans for domestic cat can be found in [26].

The domestic cat (*Felis catus*) is one of the world’s most widely kept pets. These cats possess a highly complex nasal cavity and a well-developed sense of smell, which plays important roles in feeding [15,16] and social interactions [17,18]. Cats have high olfactory acuity and disturbance to the sense of smell may cause the cat to refuse food [19,20]. Terrestrial mammals usually have three sets of bone structure, called turbinates, in their nose: naso-, maxillary, and ethmoid turbinates [21]. In the cat, these turbinates are significantly more complex than in either humans or rodents (Fig 1) and are comparable to those of the domestic dog [22,23]. The area of olfactory mucosa in the cat, housed mostly in the ethmoid turbinates toward the posterior end of the nose, is ~20 cm^2^, about four to five times that of humans and only twofold less than the average dog [24]. The complex nasal turbinate structure in the cat and the importance of its olfactory function to its survival provide an excellent model to examine the structure-function relationship. Since the domestication of its ancestor—the African wild cat (*Felis silvestris lybica*), the domestic and feral cat has spread along with human settlements and thrives in various climates. The complex nasal structure may be important for cats to adapt to these diverse environments [25].

Here, we report our development and use of an *in silico* model based on micro-CT scans and histology sections to investigate the function of the nasal structure in domestic cat, *Felis catus*. First, aided with iodine-based contrast agent, we used micro-CT to capture the delicate nasal airways with high necessary image resolution (~19 μm isotropic per pixel, see Fig 1). Histology sections then allowed us to quantify the distribution of various types of epithelia and their relationships to the airflow paths. Finally, computational fluid dynamics (CFD) modelling was used to quantitatively examine air and odor delivery speed and efficiency to the olfactory region. By comparing to existing models of rat and human, our data suggest that the ethmoid turbinates in domestic cat may function as a parallel coiled chromatograph to improve odor processing efficiency and speed. The outcome of this study raised hypotheses around what future computational and behavioral studies can be designed, which will not only help us better understand the health and well-being of the domestic cat, an important pet, but will also elucidate nasal structure-function in a broader range of mammals, including in rodents, a common prey of cats, as well as in humans.

## Results

### Morphology and Histology

**Fig 2:**
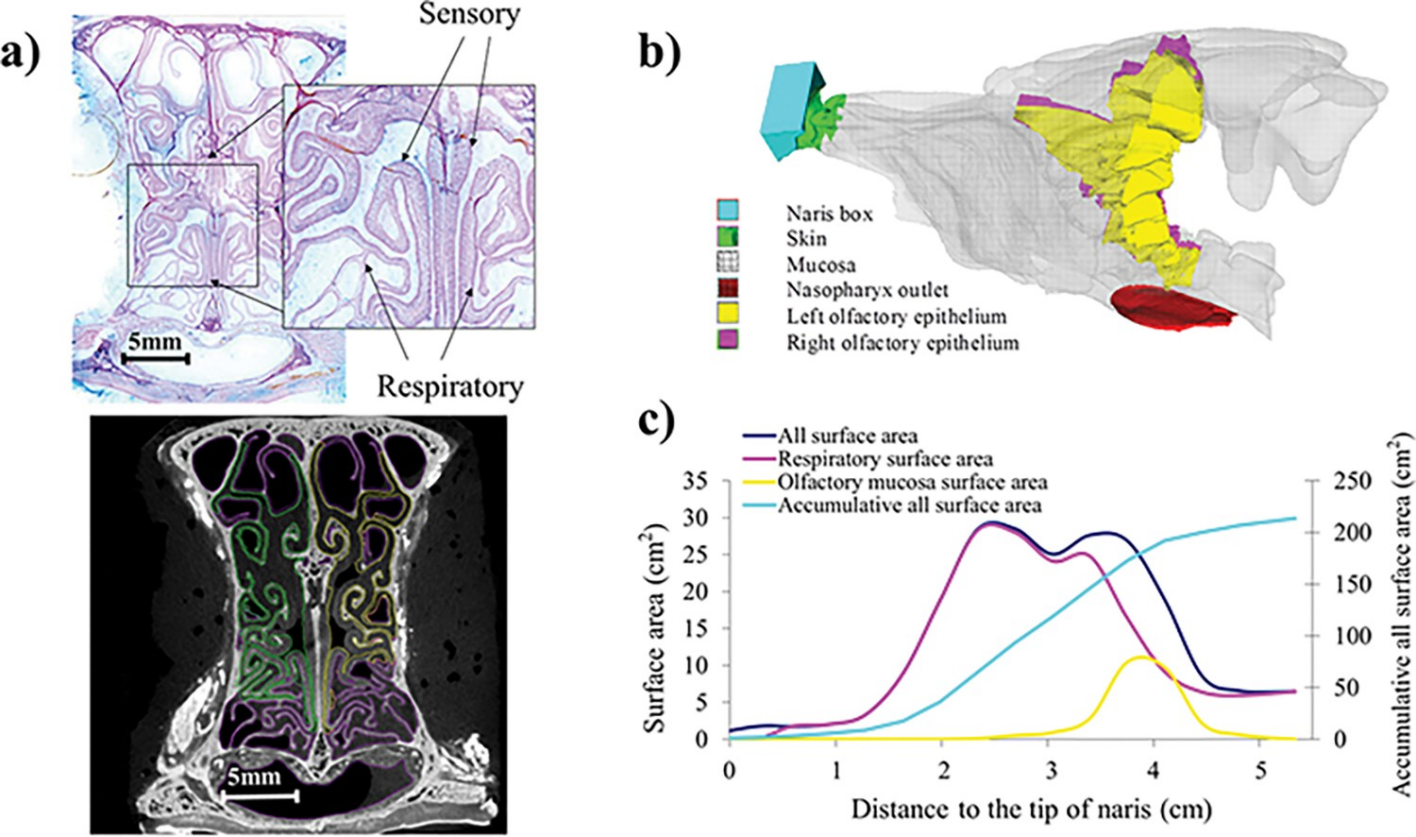
a) The distributions of various types of epithelia were determined through sequential histological sections: respiratory epithelium is thin and contains goblet cells, whereas olfactory epithelium is thicker and contains Bowman’s glands. Skin-like squamous epithelium is located in the anterior tip of the nose. Each histological section is then aligned with micro-CT images, on which locations of epithelium types are labeled based on histology features. b) The final computational model with different types of epithelia, inlet (naris box), and outlet (nasopharynx). c) The nasal surface area and percentages of various types of epithelia plotted as a function of distance to the nostril.

A domestic short hair cat cadaver was obtained, dissected, and fixed in 4% paraformaldehyde. Micro-CT scans of the head were then taken with contrast agent (see the full set of scans in [26]). A three-dimensional computational fluid dynamics (CFD) model of the nasal cavity and nasopharynx was constructed based on the micro-CT scans (Fig A in S1 File); the distributions of various types of epithelium were determined through sequential histological sections (Fig 2A and 2B; also see Methods). The surface area and percentage of various type of epithelium coverage were plotted as function of distance to the nostril (Fig 2C). Total olfactory epithelium surface area in our model is ~27.5 cm^2^, or 12.1% of total surface area, which is close to the literature-reported value [24]. The olfactory epithelium begins anteriorly in a dorsal medial passage and then extends to cover most of the posterior ethmoid region. At its peak (~39 mm from the nostrils), the olfactory epithelium comprises ~50% of the mucosa. The ethmoid turbinate extends into the frontal sinus (Fig A(e) in S1 File). This feature may serve as an additional accumulator of odor. However, we also found that not all ethmoid turbinates are covered with olfactory epithelium, especially the fronto-ethmoturbinals, and it is quite common to have one side of an ethmoid turbinate covered with olfactory epithelium and the other side (often the side further downstream) covered with respiratory epithelium (Fig 2A).

### The dorsal medial (DM) stream and separation of olfactory and respiratory airflow

**Fig 3:**
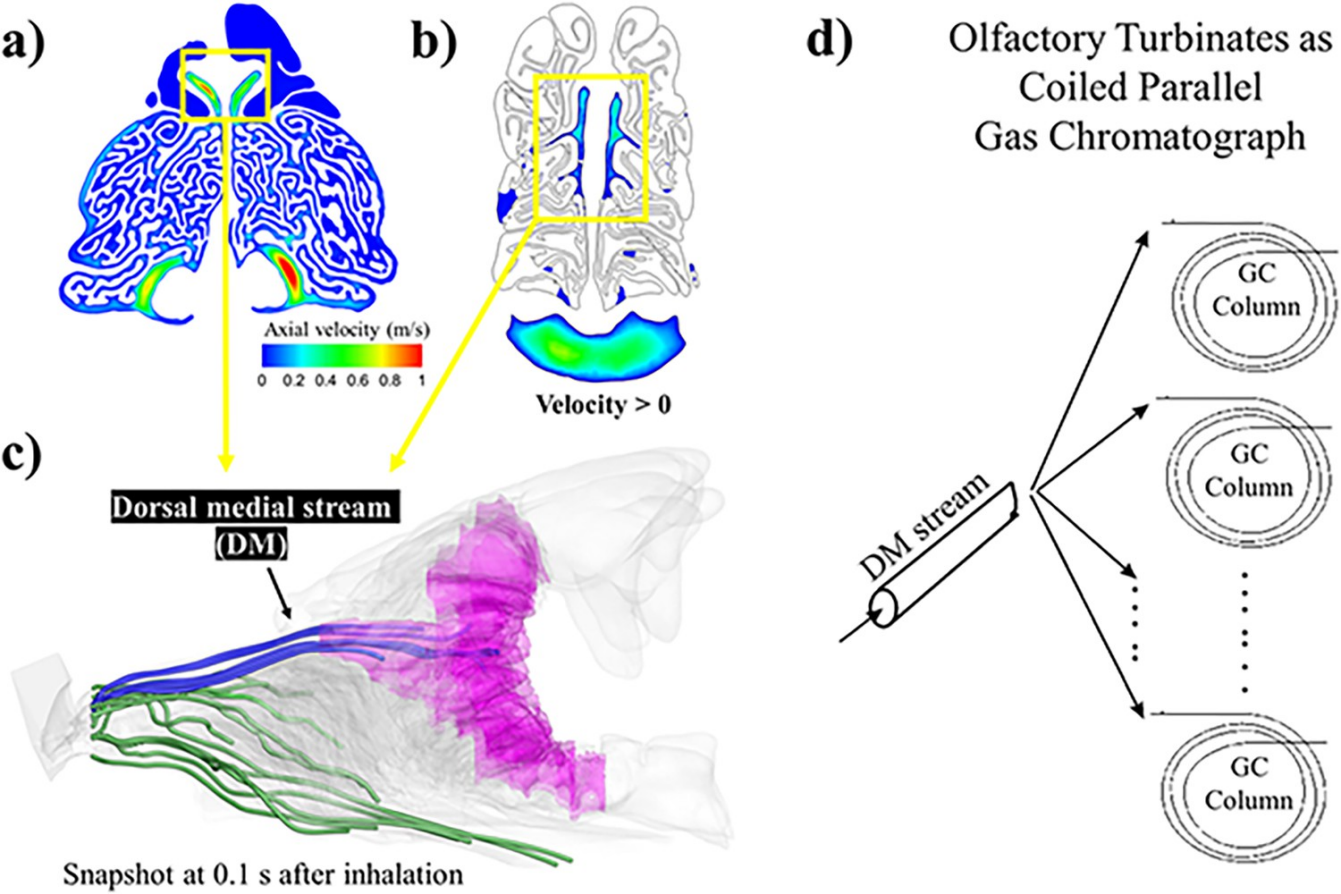
(a-c) In the cat, during inspiration, the bulk of inspired air passes through the respiratory (maxillary) turbinates (a), whereas a portion (~15–20%) enters the olfactory region (b), conveyed by the high-speed dorsal medial (DM) stream (c). Snapshot at 0.1s after inhalation showed that streamlines in the DM stream reaches deep into the olfactory region (see blue lines), faster than those spread across the respiratory turbinate region (see green lines). The axial flow component (flow in the direction from nostrils to pharynx) is positive in most of maxillary turbinate regions (a) but is positive only in the central DM region of the ethmoid turbinates. The axial flow component in the lateral ethmoid turbinate is mostly negative (no color), which indicates reversing or lateral flow. These different flow patterns suggest that respiratory turbinates branch in directions that do not block or redirect the airflow, spreading incoming airflow into parallel channels to increase heat and water exchange efficiency, whereas the olfactory turbinates are scroll-shaped, extending centrally to laterally and redirecting flow from the DM stream into lateral channels. d) Based on these observations, we hypothesize that olfactory turbinates function as a coiled parallel gas chromatograph, with the high-speed DM stream feeding airflow into parallel lateral ethmoid coils, each serving as a CG column.

**Fig 4:**
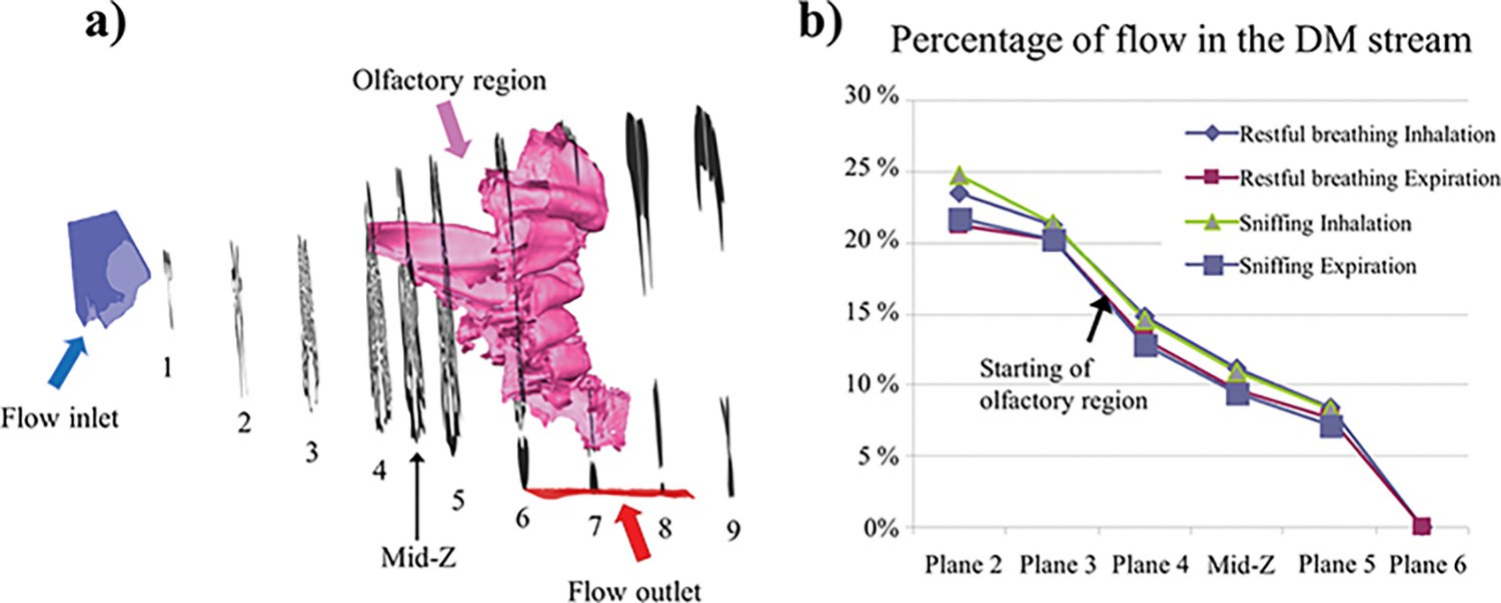
The dorsal medial (DM) air stream in cross sections of the cat nasal model (a) as the percentage of total flow rate (b), which decreases as it enters the olfactory region, where flow is diverted to the lateral regions. Airflow remains similar between restful breathing (22 ml/s) and sniffing (140 ml/s) but is slightly smaller during expiration than during inhalation.

Nasal airflow patterns were simulated using commercial CFD software under a steady restful breathing flow rate (22 ml/s) [27] (see Methods). We observed two distinct flow regions in cat nose: respiratory and olfactory flow regions. During inspiration, anteriorly, the incoming flow spreads slowly across the maxillary (respiratory) turbinate, except a very high-speed stream in the dorsal medial passage (DM, see Fig 3; see also Fig A(f) in S1 File for details), which accounts for about ~15–20% of total flow rate (see Fig 4B) that penetrates into the olfactory region, similar to what has been reported in dog [28], other felids [25] and other mammalian species [11,13,29]. The percentage of the DM stream remains similar between restful breathing and sniffing flow rates in cat (see Fig 4). No visual evidence suggested that flow within the respiratory region spreads into the DM stream. The separation of the DM stream from the rest of the air stream likely enables dual function of the nose: the bulk of inspired air passes through the respiratory (maxillary) turbinates and is thus conditioned and filtered, whereas a portion of it (~15–20% see Fig 4B), conveyed by the high-speed DM stream, bypasses the respiratory regime, and delivers the environmental odor quickly to the olfactory region with great efficiency.

Next, we examined the flow pattern differences between respiratory and olfactory flow regions by focusing on the positive axial flow component (velocity component in the direction from nostrils to pharynx; Fig 3A and 3B). Respiratory turbinates branch in directions tangent to the airflow, thus spreading the incoming airflow into parallel channels to increase heat and water exchange. As the result, the axial flow component is positive in most of the respiratory region (Fig 3A). Olfactory turbinates, in contrast, are scroll-shaped, extending centrally to laterally, thus redirecting flow from the DM stream into lateral circulating channels. The axial flow component is positive only in the central DM region and is zero or negative in lateral regions, indicating a reversing or laterally circulating flow (see also Fig B in S1 File for a complete presentation of streamlines during both inhalation and exhalation). Flow rate in the DM region gradually reduces with distance (see Fig 4), as flow is diverted to the lateral regions.

### Cat nose absorbs odorants differently than rat or human noses

**Fig 5:**
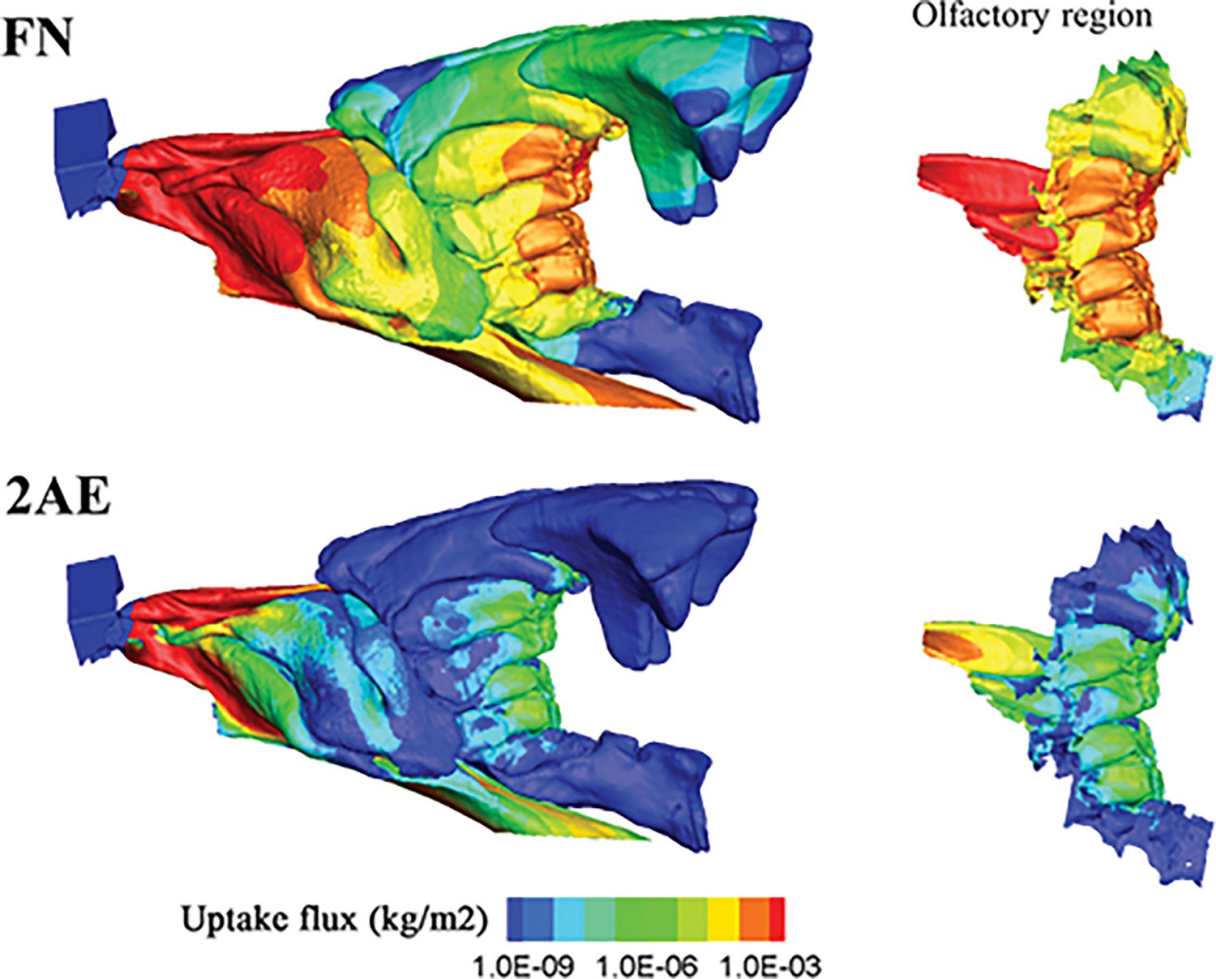
Odor absorption map of two odorants with different solubilities, 2-acetylthiazole (2AE, high) and 2-(1-mercaptoethyl) furan (FN, intermediate), at all nasal mucosal surfaces (left) and at the olfactory region only (right). The highly soluble odorant 2AE is more strongly absorbed anteriorly, depleting available molecules in air stream when reaching the posterior ethmoid region, whereas the intermediate-soluble odorant FN has optimal sorption in the olfactory region.

**Fig 6:**
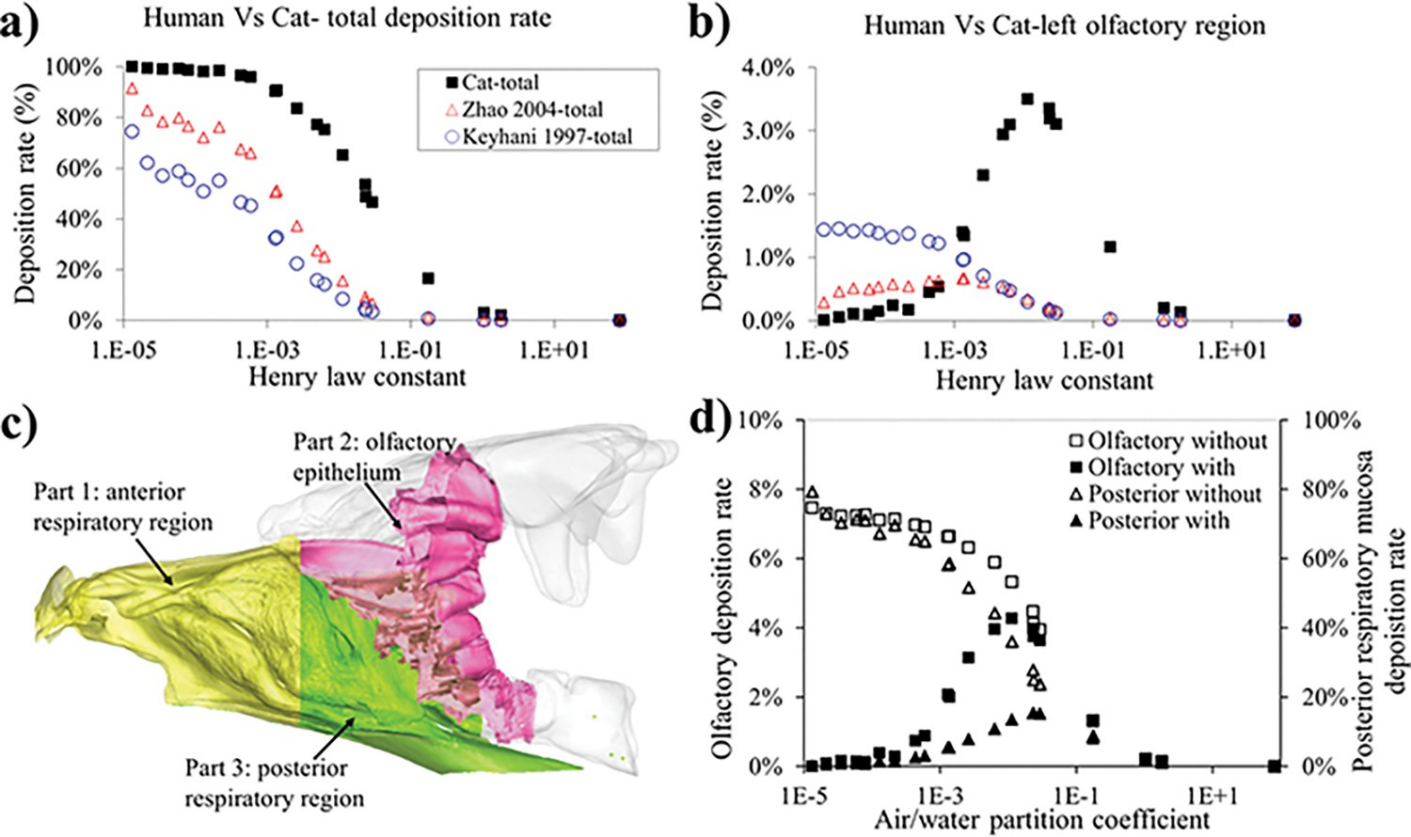
(a, b) Total nasal odor absorption (a) and absorption in the olfactory region (b) in human [[30,33]] and cat [present study]. (c) The nasal cavity is divided into three parts, anterior respiratory region, olfactory region, and posterior respiratory region. (d) Quantitative testing of the hypothesis that the dorsal medial stream allows odorous air to reach the olfactory region more efficiently by computing absorption in two scenarios: with and without absorption in the anterior respiratory region.

Next, we simulated the odor absorption onto the mucosa as the odors are transported via nasal airflow, based on published methods [30] (see Methods). Fig 5 shows the absorption map (in log scale) of two odorants under the same restful breathing scenario (22 ml/s): a very mucosa-soluble 2-acetylthiazole (2AE) and an intermediate-soluble 2-(1-mercaptoethyl) furan (FN, see Table A in S1 File for mucosa solubility and other properties). Most of compounds included in the table are typically formed during the cooking of meat or the processing of cat food, which is the main diet of the modern domesticated cat [16]. It is commonly expected that the more easily a compound is absorbed by the mucus, the more accessible it is to the olfactory neural epithelium. However, as shown in Fig 5, the highly soluble odorant has stronger absorption anteriorly, which depleted the available odorant in the air stream when reaching the posterior ethmoid region, whereas the intermediate-soluble odorant actually has higher olfactory absorption. Compared to our own published models for human and rat [31,32], cat shows significantly more efficiency in overall nasal absorption and stronger peak of olfactory absorption (see Fig 6A and 6B), but the absorption peaks occur at slightly different solubility ranges.

### The dorsal medial (DM) stream increases odor delivery speed and efficiency

To examine how the dorsal medial (DM) stream may increase odor delivery speed and efficiency, we divided the nasal cavity into three parts (Fig 6C): (1) the anterior respiratory region that leads to both the olfactory and posterior respiratory regions and includes the anterior non-olfactory portion of the DM airway, (2) the olfactory epithelium (including the posterior DM stream and most of the ethmoid turbinates), and (3) the posterior respiratory region that is parallel (lower) to the olfactory region. We then computed the airflow from the anterior respiratory region (part 1) to both the olfactory region (part 2) and the respiratory region (part 3), under two scenarios: with and without absorption in the anterior respiratory region (part 1). We would expect that the olfactory region absorption rate would be less affected between the two scenarios, due to the highly efficient delivery of the DM stream, whereas the posterior respiratory region would see greater reduction in absorption between these scenarios (see Fig 6C). The results (see Fig 6D) matched our prediction: for the olfactory region, the peak absorption is ~7.5% without anterior absorption and reduces to ~4.3% with anterior absorption; however, the absorption for the posterior respiratory region is ~79% without anterior absorption and reduces to ~15% with anterior absorption. This effect is more prominent for highly soluble odorants. This analysis also confirms the reason for the peak absorption in the olfactory region for the intermediate-soluble odorants—a tradeoff between an odorant’s mucosal solubility and upstream absorption that depletes airborne odorants prior to the airflow reaching the olfactory region. But the existence of the high-speed DM stream would reduce the impact of upstream absorption and allow more high solubility odorants to escape and reach the olfactory region, compared to without the DM stream, i.e., posterior respiratory region (see Fig 6C).

In comparison, we also found a similar effect for the rat: upstream absorption affects its olfactory region absorption less than the posterior respiratory region (see Fig C in S1 File: olfactory region, 26.4% vs 19.6%; posterior respiratory region, 79.0% vs 43.6%) but not for human (olfactory region, 4.4% vs 1.8%; posterior respiratory region, 50.4% vs 27.9%). The simpler turbinate structure in humans does not seem to provide a DM stream for high-efficiency olfactory delivery nor does it provide high overall odor absorption.

We then simulated airflow streamlines by releasing neutral-buoyant particles at the nostril and tracing their trajectory (see Fig 3C). We created a snapshot at 0.1 s after the particles were released, which shows that streamlines in the DM stream already reach deep into the olfactory region, much faster than those spread across the respiratory turbinate region, benefiting from the high-speed DM stream. Together, these results suggest that in cat the DM stream provides efficient, speedy odor delivery to the olfactory mucosa.

### Cat olfactory region functions as parallel packed gas chromatography system

The form and shape of the ethmoid turbinates in several mammals appear to be quite similar (e.g., dog, rat, cat), which we hypothesize might be an important evolutionary advance in what we would term “parallel coiled gas chromatography”. In chromatography theory, a longer gas chromatography (GC) column can increase retention of odors, but for the nose, the physical size limitation of the head prevents nasal channels from continuing to increase as a straight tube; thus, coiling them as turbinate channels is a good option. But if all of the DM flow is connected to only one olfactory coil, the high flow rate would wash out the odor too fast. To some extent, lower flow rates benefit GC efficiency, but lower flow rate would also result in fewer total odorant molecules per breath and longer latency for odors to reach olfactory receptors, which is not ideal for odor detection. Thus, stacking these coils in parallel, with a high-speed high-flow DM stream as the main supply of flow, would lower flow rate within each coil while maintaining a high overall olfactory flow rate (Fig 3D).

**Fig 7:**
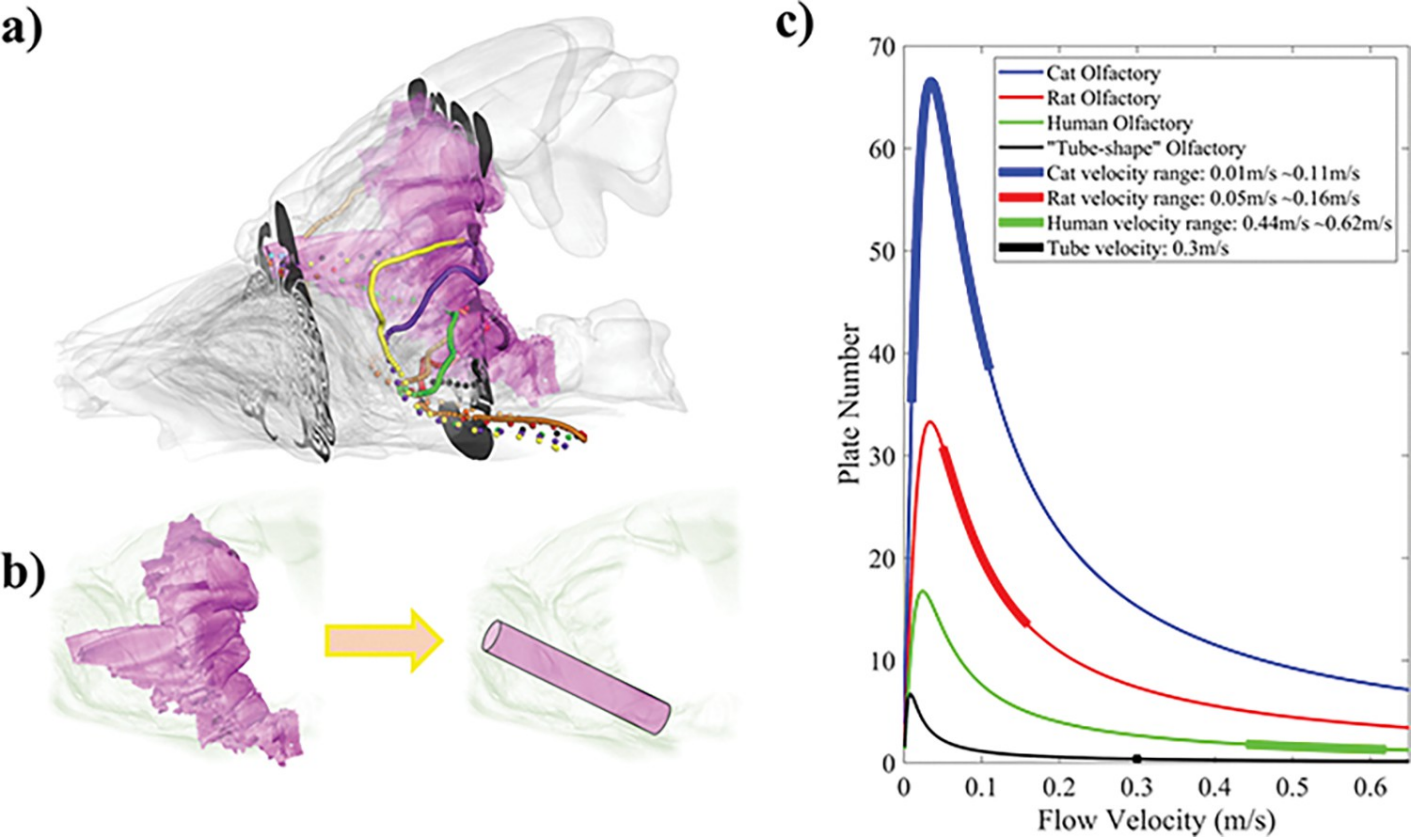
(a) A snapshot of parallel trajectories of odor particles in the cat olfactory region (colored dots). (b) Comparison of GC efficiency between cat coiled (left) and amphibian-like “straight tube” (right) olfactory regions of the same length based on plate number theory (c) The calculated plate number for olfactory regions of cat (blue curve), rat (red curve), human (green curve), and the straight “tube” (black curve). The thickened lines represent the actual airflow velocity range in the olfactory regions of the different species.

To test these hypotheses, we evaluated the GC efficiency of the olfactory regions of various species using theoretical GC plate number (see Methods), which is a widely adopted concept to describe the GC efficiency [34,35]—a higher plate number represents higher capability to differentiate different odorants into different absorption peaks. As shown in Fig 7C, the parallel coiled GC model of cat (blue curve) has a peak plate number N_peak_ = 67. We then simulated an amphibian “straight tube” olfactory system of the same length for comparison (Fig 7B), which resulted in N_peak_ = 7 (Fig 7C, black curve). Furthermore, the computed airflow velocity in the cat olfactory region is sufficiently low (0.01–0.11 m/s; Fig 7C, blue dots) due to the parallel flow paths to achieve the highest plate number of the system. In contrast, the velocity in a “straight tube” olfactory system fed directly from the DM stream (~0.3 m/s; Fig 7C, black dots) is far too high to achieve the optimal plate number, which further reduced the actual plate number to that of less than ~1/100^th^ of cat olfactory region. The results indicate that the complex cat olfactory region provides higher GC efficiency in processing different odors than a simple “straight tube” system.

We computed the plate number curves for the rat (Fig 7C, red curve) and human olfactory region (Fig 7C, green curve), which resulted in peak plate number of N_peak_ ≈ 33 and N_peak_ ≈ 17; respectively. More importantly, while the airflow velocity in the olfactory region of the rat is sufficiently reduced to achieve near-optimal plate number, the airflow velocity of the human olfactory region, which also lacks the “parallel GC” feature, is too high to achieve optimal plate number (Fig 7C, red vs. green thickened line), with an actual plate number of only ≈ 2.

## Discussion

Based on a high-resolution anatomically accurate computational model, we characterized and quantified key characteristics of nasal airflow patterns in the domestic cat (*Felis catus*) that reaffirms the importance of some common nasal aerodynamic features among mammalian species: the separated respiratory and olfactory flow regions and the occurrence of the dorsal medial (DM) stream (e.g. in rodents [36,37], dog [22], and bobcat [25]). The result compared to the simulation performed in bobcat is remarkably similar. The separation of olfactory and respiratory flow paths is not completely based on ethmoid versus maxillary turbinates and a substantial amount of respiratory airflow passes over the anterior fronto-ethmoturbinals (see Fig 7 in Pang el al [25]). This affirms the importance of using accurate histology information as in the current study to avoid overestimating the olfactory epithelial coverage based purely on ethmoid turbinal surface area. Related, Eiting et al [29] studied 6 species of bats of different ecologies with likely different olfactory reliance and reported that while the olfactory (DM) stream seems to be conserved across species, there was no clear difference in either DM stream speed and the patterns of airflow through the olfactory region among species. This is interesting, but as we showed in our study, the airstream speed is not linear nor the only indicator of the olfactory system efficiency and visual inspection can only reflect large qualitative differences. Certainly, the forming of DM stream is not universal, Smith et al. studied airflow in a basal primate lineage [13], and found that for slow loris, a species that has some adaptations for improved olfactory acuity, while there is some segregation of respiratory and olfactory flow, it not as distinct as the DM stream in other well-studied mammals (e.g., dog). These inconsistent, but largely descriptive findings, prompted the need for new quantitative analysis tools to investigate the functional relevance of the DM stream as well as olfactory flow patterns.

Here, we attempted to investigate the nasal structure-function relationship using cat nose as a model with gas chromatography theory based new quantitative analyses. First, we quantitatively confirmed that the DM stream in cat nose does increase efficiency of odor delivery to the olfactory region with fast speed, and that airflow in the lateral coiled ethmoid turbinate region is mostly recirculation originating from the DM stream. Second, we hypothesize that ethmoid turbinates may function as a coiled parallel gas chromatograph (GC). Earlier studies that examined the hypothesis of olfactory chromatography were based mostly on amphibians (e.g., frog [5], salamander [38]), whose nasal cavities are mostly a simple tube or cavity, without turbinates. In chromatography theory, a longer GC column is often advantageous as it increases retention of odors. However, confined by the physical size limitation of the head, nasal channels cannot increase in length infinitely as a straight tube, so coiling them as turbinate channels is a natural option, similar to how GC columns are packed in reality. And this is also remarkably similar to what is seen in a different sensory organ, the cochlea. A long coiled, snail-like cochlea is unique to mammals, while in birds and in other non-mammalian vertebrates, the inner hearing organ, despite being called “cochlea”, is not coiled and is instead a blind-ended tube. The coiled cochlea unique to mammals is an evolutionary advance responsible for our extended frequency range due to the longer tube for additional octaves [4]. In comparison, the olfactory turbinates composed of the coiled ethmoid turbinates may be a similar structural adaptation from vertebrates to mammals to enhance olfactory performance. There is evidence that the evolutionary occurrence of complex ethmoid turbinates in the mammalian nose correlates to improved olfaction performance [9].

In addition to a long column, an efficient GC needs an optimal airflow velocity. The higher the linear velocity, the faster the analysis but may lower the separation resolution between different chemicals. A typical capillary column for modern GC is often 10–50 m long, with optimal linear velocity of ~0.2–0.6 m/s [39]. For mammalian ethmoid turbinates, this presents additional conflict: if all the DM flow is connected into only one olfactory coil, the high airflow velocity would wash out the odor too quickly, but a lower DM flow rate would result in less total odorant molecules per breath and longer latency for the odor to reach the olfactory receptors, which also is not ideal for odor detection. Thus, stacking these coils in parallel, with a high-speed high-flow DM stream as the main supply of flow, would maintain high overall olfactory flow rate while lowering airflow velocity within each coil (see Fig 3D). Based on GC plate theory, we estimated that the parallel structure of the cat ethmoid turbinates can achieve a theoretical plate number more than 100 times higher achieved by an amphibian-like, single straight olfactory tube occupying a similar physical dimension, taking advantage of both longer coiled paths and optimal airflow velocity. Thus, the forming of complex parallel ethmoid coils is likely a key feature that enhanced olfactory performance among mammals, similar to the mammalian cochlea in hearing. During the comparison, we noticed that in the human olfactory region, which lacks the parallel GC feature present in cat, the airflow velocity is too high to achieve optimal plate number. So, potentially too high olfactory airflow speed does not necessarily benefit the olfactory system, but this conjecture needs to be tested broadly across other species in the future.

Another consideration based on the GC analogy is the detector—in this case, the olfactory receptors. In vertebrates such as salamander, with a simple cavity, the olfactory receptors were expressed as bands perpendicular to the flow path, forming a linear-array detector [38]. With the GC column coiled in mammals, we would expect olfactory receptors to be located at roughly equal locations in each coil, forming a ring-shaped detector array, so that the signal can potentially converge at both spatial and temporal domains. The distribution of olfactory neurons is unknown for cat at present, but the zonal olfactory distribution patterns in rodents do seem to form ring shapes centered on the DM stream [14,40]. Our recent work further suggested that the intrinsic olfactory epithelial response profile may be tuned by the sorption profile as defined by the nasal airflow and GC separation [41].

For cat, during expiration, less flow enters the DM stream and olfactory regions; for example, in Fig 4B, at the mid-Z plane 11.5% of the nasal airflow enters the olfactory recess during inhalation versus 9.5% for exhalation, a 2% reduction for exhalation. This feature differs from that reported in dog, where no appreciable airflow enters the olfactory recess during exhalation [22], but is similar to that reported in rat [42,43]. There has been functional evidence that the rat does have retronasal olfaction (i.e., odorous air enters the nasal cavity during exhalation), albeit less intense than the orthonasal olfaction [44].The expiratory air stream in cat provides some aerodynamic basis for this species to have retronasal olfaction. Similar to that proposed for rat [42,43], the reduced airflow during exhalation in cat may induce less washout of the odorant from the olfactory region, retaining odorant longer in the ethmoid air spaces. The comparison between cat and rodents may be of ecological interest, as cats prey on rodents. We found that, compared with rat, cat shows significantly more efficiency and stronger peak in nasal absorption, but the absorption peaks occur at different solubility ranges. We believe it will be interesting to validate (e.g., through behavioral studies) whether cat may attune to odorants differently than do rat and human. How structure-function differences affect perception and behavior across species is certainly another area warranting further investigation.

## Conclusion

This study employed interdisciplinary techniques (high resolution microCT, sequential histological sections and staining) to create an anatomically accurate computational fluid dynamics nasal model for a domestic short-hair cat. Based on the new analysis of nasal airflow and odor transport, we revealed that their ethmoid olfactory turbinates may function as a “parallel coiled gas chromatograph”, that significantly improves the gas chromatograph efficiency more than 100 times, compared to an “amphibian-like” straight channel fitting a similar skull space. Domestic cats, one of the world’s most popular pets, co-exist within and alongside human settlements, and thrive in various environments. The understanding of its well-developed sense of smell that plays a vital role in their food selection and social interactions is important to understand their successful adaptation to these diverse environments, as well as contribute importantly to our understanding of their health and well-being.

## Methods

### Ethics statement

The experiments performed were approved by the Waltham Animal Welfare and Ethical Review Board (AWERB) and the University of Pennsylvania Institutional Animal Care and Use Committee (IACUC #802080).

### Sample

A Domestic short hair, adult, male, approximately 5.4 kg of weight, was obtained from the Educational Memorial Program at the MJ Ryan Veterinary Hospital of the University of Pennsylvania–a program where owners donate their deceased pet’s remains for educational purposes. This animal died from an accident unrelated to the head or nasal sinus. The head was subsequently removed from the cadaver, cleaned of fur, skin, muscle etc., fixed in 4% para formaldehyde for 1 week and then transferred to Phosphate Buffered Solution (PBS).

### Micro CT imaging

Prior to finalizing the imaging protocol, several short pilot scans were run with various techniques: 1) a clinical CT at PENN Vet Hospital with an in-plane resolution of 130 um and between plane resolution of 500 um (130x130x500 um) under a different IACUC protocol (#802001). The resulting images (see Fig A(a) in S1 File) did not provide sufficient resolution to capture the anatomical feature; 2) a scan using microCT without contrast enhancing dye (Fig A(b) in S1 File), which also lacked sufficient clarity. CT normally produces a sharp contrast between air versus soft tissue, but a low contrast between tissue versus water-based buffer solution. However, completely removing buffer solution and drying the tissue prior to the scan would result in significant tissue shrinkage and damage during a typical microCT scan that lasts about 6–10 hr, and the sample would then be unsuitable for any subsequent histology usage.

One outcome from our trials is the successful use of an iodine dye (25% Lugol solution) to enhance the contrast between airway soft tissue versus buffer solution (see Fig A(c) in S1 File). The dilution of the Lugol solution matches the osmolality of the plasma to reduce tissue shrinkage. It was subsequently determined that 1 week submerged with the dye would be optimal for the dye to penetrate the tissue fully. Prior to the final scan, the tissue was then removed from the dye and rinsed with Phosphate-buffered saline (PBS), wrapped and sealed with parafilm (Parafilm Nursery Grafting Tape-Oesco, Inc.) before scanning, without completely draining all the solution. The position/angle of the specimen placement was carefully adjusted and tilted to fit the nasal structure with in the 45 mm diameter field of view, the widest view of the scanner (Viva CT 40 microCT scanner, Scanco USA, Inc). The final scan took approximately 10 hr and generated a total of 3,300 images, each with 2048x2048 pixels and an isotropic resolution of 19 μm per pixel (see the full set of scans in [26]). We analyzed the images afterwards and identified that the thinnest nasal airway in cat is around 100 um, which indicated that a high resolution imaging modality was necessary to capture the nasal airway accurately. Various X-ray scanner settings (e.g., X-ray bulb voltage and current) were tested prior to finalizing the optimal combination of parameters (70 kv, 114 uA), based on the image quality. A 3D median filter with a mask of 5x5x7 was applied to remove image noise and preserve the edge that interfaced between airway and mucosa (see Fig A(d) and A(e) in S1 File). We also tested various other filters: low pass, band pass, anisotropic diffusion filters, and determined that the 3D median filter and this particular mask setting performed the best.

### Histology

After the microCT scan was completed, the specimen was then decalcified with 3.5% Hydrochloric acid and 0.1% Ethylenediaminetetraacetic acid tetrasodium (HCL-EDTA) solution for 48 hr and cryoprotected with a series of ascending sucrose solutions (10%, 20%, 30% sucrose in PBS) for 24 hr each, embedded in Optimal cutting temperature (OCT) compound and sequentially sectioned in 20 μm thickness in a cryostat. The sections were stained with alcian blue (pH 2.5, to stain mucus and goblet cells) and neutral red (to stain nuclei) and cover-slip applied. The histology sections served to differentiate different types of epithelium. Under microscopy, the skin-like squamous epithelium is located in the anterior end of the nose, respiratory epithelium is thin with goblet cells (blue), whereas olfactory epithelium is thicker and contains Bowman’s gland. The histology image was aligned with the microCT images manually, and the distribution of the three epithelium types were labeled in our final model (see Fig 2).

### Model construction

The three dimensional anatomically-accurate model of the cat nasal cavity and nasopharynx was constructed using the AMIRA software (Visualization Sciences Group, Burlington, MA) based on the segmentation of the microCT images [31]. A second commercial software package, ICEM (Ansys, Inc., Canonsburg, PA) was then used to generate the nasal airway meshes using tetrahedral elements. Then, the boundary-adaptive mesh refinement feature of FLUENT (Ansys, Inc., Canonsburg, PA) was further used to refine cells in the proximity of the nasal wall in order to resolve the boundary layer where odorant mass transport mostly occurs (see Fig D in S1 File). Grid independence was checked by increasing the number of the mesh (Fig D in S1 File). Between grid refinement from 22 million to 80 million mesh size, the changes in the velocity and pressure fields were found to be minor. Hence throughout the study, results from a 40 million grid are presented. A box is also created near the nostrils to enclose the surrounding air and skin surface, to more accurately capture the external air flowing into the nostril [32,43]. Previously published rat [32,43] and human [31] nasal CFD models were used for cross species comparison.

### CFD simulation

The following assumptions are adopted in the CFD simulation 1) nasal airflow is quasi-steady, laminar, incompressible, and Newtonian. The effects of unsteadiness on the developing flows can be estimated by the Womersley number *W*_0_ [45] and the Strouhal number *S* [46]:

$$W_{0}=R\sqrt{\left(\frac{2\pi f}{\nu }\right)},S=\frac{2\pi fL}{U}$$ (1)

and the effect of turbulence can be estimated by the Reynolds number:

$$Re=\frac{UR}{\nu }$$ (2)

where *R* is the hydraulic diameter of the external naris, *f* is the cycling breathing frequency, *ν* is the air kinematic viscosity, *L* is the axial length along the nasal cavity, and *U* is the average velocity. The Womersley number is a dimensionless ratio of the unsteady inertial forces in relation to viscous forces. Whereas the Strouhal number is derived from the ratio of the steady boundary layer thickness to the Stokes layer thickness, representing the ratio of unsteady inertial forces or local acceleration to the convective inertial forces. The flow rate and restful breathing frequency *f* of domestic cat, is determined by scaling of experimental data [27], to be about 22 ml/s and 1 Hz based on a 5.4 kg body weight. Based on the obtained nasal airway dimension through microCT and CFD modeling, the *W*_*0*_, *S* and *Re* number is calculated and plotted in Fig E in S1 File as a function of axial distance to the nostril. In general, a quasi-steady assumption is valid when *W*_0_ is less than 4 and *S* is less than 1 [32,47], and a laminar assumption is valid when *Re* < 2300. Thus, for cats, the unsteady and turbulent effect is negligible at restful breathing (Fig E in S1 File) but may occasionally exceed the limit at higher sniffing flow range. The sniffing flow rate and frequency is estimated to be 140 ml/s and 5 Hz [22,23,27]. While the current study focused on restful breathing in cats, which is biologically relevant as the animal often detects a novel odor during restful breathing state before switching to sniffing, we plan to further examine the unsteady and turbulent effect in cat nose in the future, similar to what we have published for rat [32,42].

2) We further assumed that the nasal wall is rigid and smooth, with no slip condition (zero velocity), and a static nostril with no movement. These assumptions were routinely applied in studies of nasal airflow in rat and human [31,32,41,43]. Atmospheric pressure was set at the inlet (the external air), while a negative pressure (outlet) was defined at the outlet (the nasal pharynx).

### Odor transport

We further simulated a wide range of odorants to understand the transport phenomena in cats that may be relevant to their olfactory function based on the calculated airflow field and the estimated physico-chemical properties in both the air phase and in mucosa [30]. Briefly, the odorant concentration at the boundary of the air/mucus interface satisfied:

$$\frac{\partial C^{\prime }}{\partial y^{\prime }}+KC^{\prime }=0\;with\;K=\frac{d_{in}D_{m}}{D_{a}\beta d}$$ (3)

where *C’* is the normalized odorant concentration, *d*_*in*_ is the hydraulic diameter (4×area/perimeter) of the nostril, *D*_*m*_ is the odorant diffusion coefficient in mucosa, *β* is the air/mucus odorant partition coefficient (defined by the ratio of odorant concentration in air phase to the concentration in the mucus at the air/mucus interface), *d* is the thickness of the mucosal layer and also the length of the path that the odorant molecules need to diffuse through, and *D*_*a*_ represents the diffusivity of the odorant molecules through air which could be determined by the Wilke-Chang equation [48]. The simulation of nasal airflow and odor absorbance has been previously validated against experimentally measured data [33,49].

The numerical solutions of the above simulation were carried-out with the commercial fluid dynamics software FLUENT (ANSYS, Inc., Canonsburg, PA). All the equations were discretized by the second-order upwind scheme for spatial dimensions. Pressure and velocity correlations were solved using the SIMPLEC method. The converged solutions were assumed to be obtained when the scaled residuals of continuity and momentum equations were less than 10^−5^. The global quantities such as flow rate at the outlet and averaged concentration of particles and odorants at certain coronal sections were also monitored to check the convergence.

### Theoretical analysis of Gas Chromatography efficiency

We hypothesized that cat olfactory turbinates may function as a coiled parallel gas chromatograph as shown in Fig 3D. A parallel GC system benefits from its multi-columns which can increase the chemical information obtained in a given time [50]. As the DM stream is separated into multiple paths through the coiled ethmoid turbinate channels, each of the parallel paths can be treated as a GC column. While it is impossible to compute every path, we took a sample of 10 paths (see Fig 7A) and averaged them to obtain an average GC length. The mobile phase is the airway in the paths, and the stationary phase is the mucus layer, assumed to be 30 μm thickness [51], coated on the inner wall of each column.

The theoretical plate concept was adopted to describe the efficiency of the GC column. We used the Golay equation [52,53] to evaluate the plate height (*H*) of the GC systems.

$$H=\frac{2D_{a}}{u}+\frac{2k^{\prime }d^{2}u}{3{\left(1+k^{\prime }\right)}^{2}D_{m}}+\frac{(11k^{\prime 2}+6k^{\prime }+1)d_{c}^{2}u}{96{\left(1+k^{\prime }\right)}^{2}D_{a}}$$ (4)

where *u* is the averaged linear velocity, *d*_*c*_ is the averaged channel width of the column, and *k*′ is the capacity factor or retention factor, which can be calculated by using Eq 5 [54].

$$k^{\prime }=\frac{C_{m}}{C_{a}}*\frac{V_{m}}{V_{a}}$$ (5)

here, *V*_*m*_ and *V*_*a*_ are the volumes of the stationary and mobile phases. *C*_*m*_ and *C*_*a*_ are the molar concentrations of the odor in the stationary phase and mobile phase; respectively. Two planes were selected (Fig 7A) to acquire the inlet and outlet concentration, *C*_*in*_ and *C*_*out*_.


$$C_{m}=C_{in}-C_{out},\;C_{a}=C_{out}$$ (6)

The plate number (*N*), which is the most important index to reflect the efficiency of a gas chromatograph system, can be calculated based on the plate height (*H*) and the column length (*L*_*c*_) [35].


$$N=L_{c}/H$$ (7)

The column length (*L*_*c*_) used in this simulation is the average length of the paths which the odor compound travelled from the olfactory inlet to the olfactory outlet (Fig 7A).

To compare to the efficiency of different olfactory systems, we conducted the same simulation procedures on a human, a rat, and a “straight-tube” model. The diameter of the “straight-tube” model was determined based on the cat olfactory DM stream inlet area, and the length of the tube is the distance between the olfactory inlet and outlet as shown in Fig 7B, so it can be assumed that the DM stream extends with the same diameter all the way to the pharynx. We compared the plate number curves of each model as a function of airflow velocity, and the comparison plots are shown in Fig 7C.

## Supporting information

S1 File Supplementary figures (Fig A—E). **Fig A.** (a-d) demonstrated different trials of CT imaging technique: (a) a clinical CT scan, field of view 6 cm (diameter), resolution 130x130x500 um; (b) high resolution microCT (isotropic 19 um) but without contrast agent, (c) microCT image with 25% lugol solution as contrast agent (Viva CT 40 microCT scanner, Scanco USA, Inc), (d) image of (c) after 3D 5x5x7 median filter. (e) The final microCT image sequence after smoothing at (labeled) distance (mm) from the tip of the nose. (f) sections of the final nasal model and simulated inspiratory velocity contours plots during restful breathing at the same axial plan as (e). **Fig B.** Streamlines between naris and nasopharynx during (a) inhalation (b) Exhalation. **Fig C.** Computing absorption in two scenarios: with vs without absorption in the anterior respiratory region of rat and human. **Fig D.** Grid independence was checked by increasing the number of the mesh. Between grid refinement from 22 million to 80 million mesh size, the changes in the velocity and pressure fields were found to be minor. Hence throughout the study, results from 40 million grid will be presented. **Fig E.** (a) Distribution of W_0_ numbers during restful breathing (15Pa, frequency = 1 Hz) and sniffing (45Pa, frequency = 5 Hz). (b) Distribution of Reynolds and Strouhal numbers during sniffing (45Pa, frequency = 5 Hz). **Table A**. Name and chemical structure of the odorants used in this study, as well as their physical parameters.
(PDF)
