# Supplementary material for: Domestic cat nose functions as a highly efficient coiled parallel gas chromatograph
Source: PLoS Comput Biol. 2023 Jun 29;19(6):e1011119. doi: 10.1371/journal.pcbi.1011119 (PMC10309622; doi:10.1371/journal.pcbi.1011119)
Supplement: S1 File — Supplementary figures (Fig A—E). Fig A. (a-d) demonstrated different trials of CT imaging technique: (a) a clinical CT scan, field of view 6 cm (diameter), resolution 130x130x500 um; (b) high resolution microCT (isotropic 19 um) but without contrast agent, (c) microCT image with 25% lugol solution as contrast agent (Viva CT 40 microCT scanner, Scanco USA, Inc), (d) image of (c) after 3D 5x5x7 median filter. (e) The final microCT image sequence after smoothing at (labeled) distance (mm) from the tip of the nose. (f) sections of the final nasal model and simulated inspiratory velocity contours plots during restful breathing at the same axial plan as (e). Fig B. Streamlines between naris and nasopharynx during (a) inhalation (b) Exhalation. Fig C. Computing absorption in two scenarios: with vs without absorption in the anterior respiratory region of rat and human. Fig D. Grid independence was checked by increasing the number of the mesh. Between grid refinement from 22 million to 80 million mesh size, the changes in the velocity and pressure fields were found to be minor. Hence throughout the study, results from 40 million grid will be presented. Fig E. (a) Distribution of W0 numbers during restful breathing (15Pa, frequency = 1 Hz) and sniffing (45Pa, frequency = 5 Hz). (b) Distribution of Reynolds and Strouhal numbers during sniffing (45Pa, frequency = 5 Hz). Table A. Name and chemical structure of the odorants used in this study, as well as their physical parameters. (PDF) [file pcbi.1011119.s001.pdf]

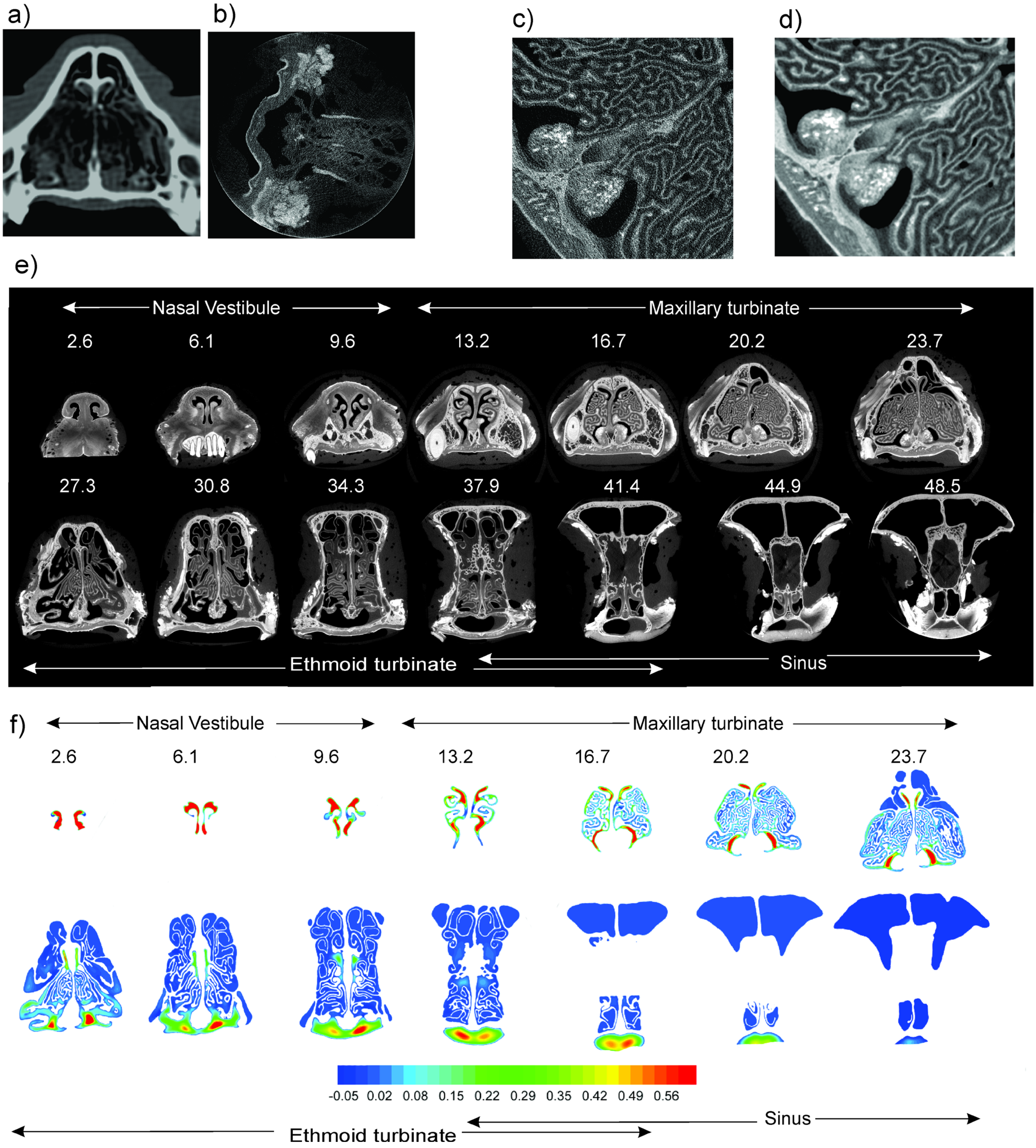

Fig A. (a-d) demonstrated different trials of CT imaging technique: (a) a clinical CT scan, field of view 6 cm (diameter), resolution 130x130x500 um; (b) high resolution microCT (isotropic 19 um) but without contrast agent, (c) microCT image with 25% lugol solution as contrast agent (Viva CT 40 microCT scanner, Scanco USA, Inc), (d) image of (c) after 3D 5x5x7 median filter. (e) The final microCT image sequence after smoothing at (labeled) distance (mm) from the tip of the nose. (f) sections of the final nasal model and simulated inspiratory velocity contours plots during restful breathing at the same axial plan as (e).

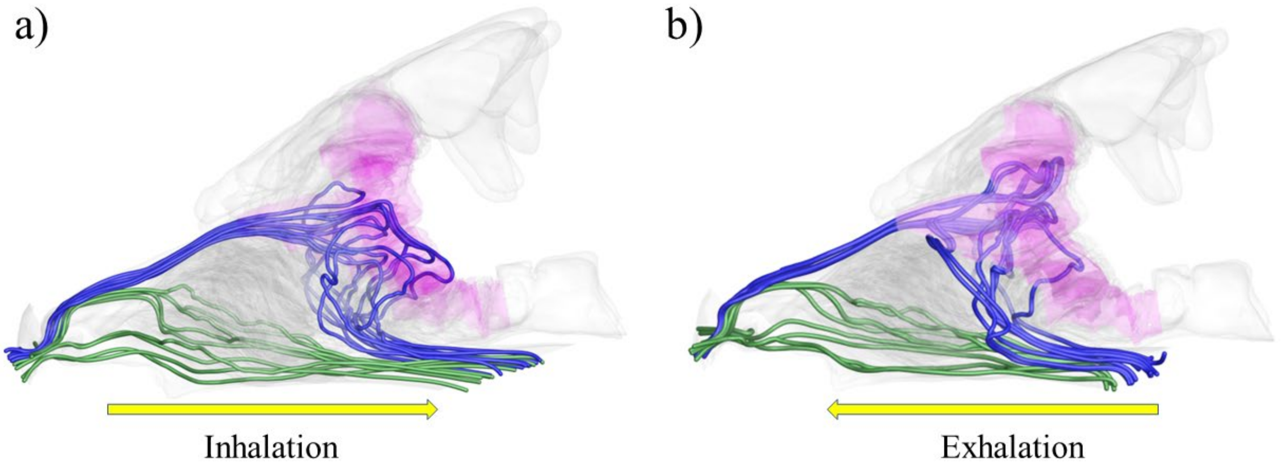

Fig B. Streamlines between naris and nasopharynx during (a) inhalation (b) Exhalation.

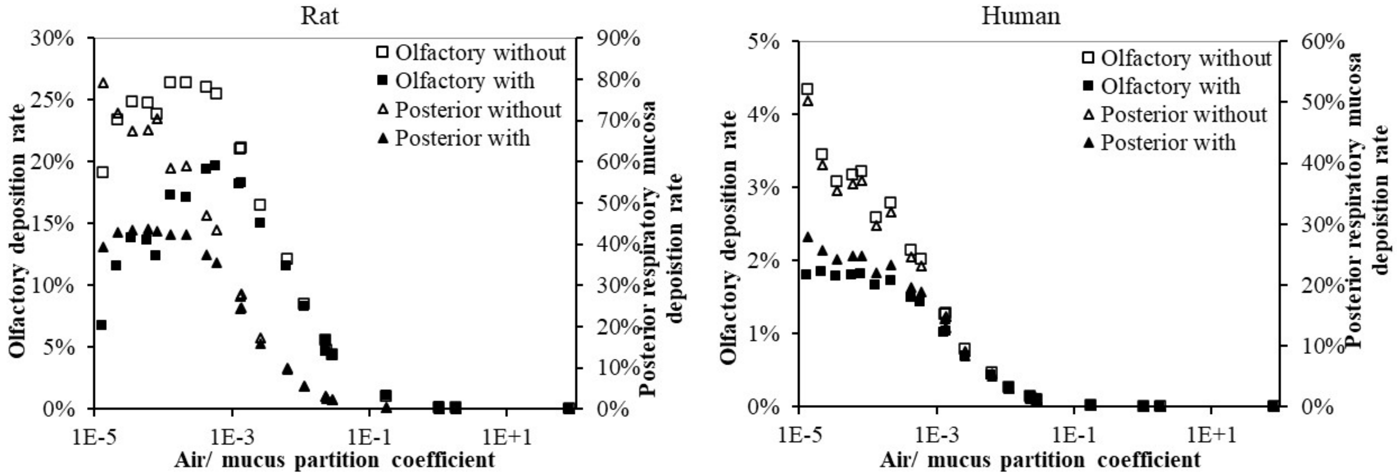

Fig C. Computing absorption in two scenarios: with vs without absorption in the anterior respiratory region of rat and human.

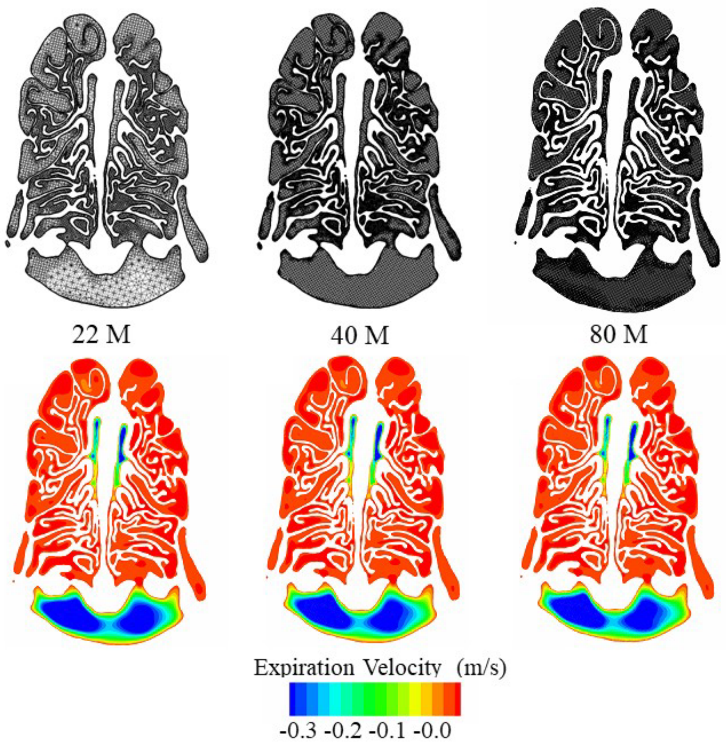

Fig D. Grid independence was checked by increasing the number of the mesh. Between grid refinement from 22 million to 80 million mesh size, the changes in the velocity and pressure fields were found to be minor. Hence throughout the study, results from 40 million grid will be presented.

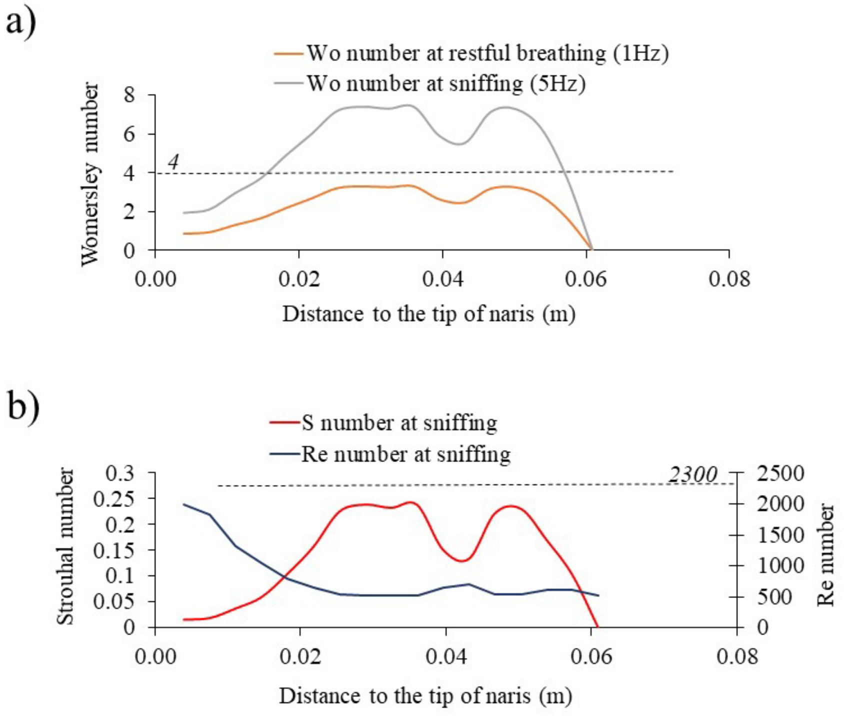

Fig E. (a) Distribution of W_0 numbers during restful breathing (15Pa, frequency=1 Hz) and sniffing (45Pa, frequency = 5 Hz). (b) Distribution of Reynolds and Strouhal numbers during sniffing (45Pa, frequency = 5 Hz).

Table A. name and chemical structure of the odorants used in this study, as well as their physical parameters.

Abbr.	Odor name	CAS number:	Molecular formula:	Molecular weight (g/ mol):	D_a , Air phase diffusivity (m^2/s)	D_m , Mucus phase diffusivity (m^2/s)	Henry's Law constant (atm-m3/mole)	LogP
2AE	2-acetylthiazole	24295-03-2	C5H5NOS	127.16	8.21E-06	8.30E-10	6.98E-09	0.67
BA	Butanoic acid	107-92-6	C4H8O2	88.11	8.82E-06	1.03E-09	5.35E-07	0.79
NAA	Nonanoic acid	000112-05-0	C9 H18 O2	158.24	5.91E-06	6.25E-10	1.62E-06	3.42
ML	Methional	3268-49-3	C4H8OS	104.17	8.24E-06	9.35E-10	5.37E-06	0.41
MT	Menthol	000089-78-1	C10 H20 O1	156.27	5.93E-06	6.35E-10	1.52E-05	3.4
2HPT	2-Heptanone	000110-43-0	C7 H14 O1	114.19	7.01E-06	7.44E-10	1.69E-04	1.98
FN	2-(1-mercaptoethyl) furan	96631-04-8	C6H8OS	128	7.68E-06	8.26E-10	2.79E-04	2.27
OC	Octanal	000124-13-0	C8 H16 O1	128.22	6.54E-06	6.91E-10	5.14E-04	2.78
MH	Methyl Heptanoate	000106-73-0	C8 H16 O2	144.22	6.71E-06	7.12E-10	7.23E-04	2.83
EUG	Eugenol	97-53-0	C10H12O2	164.2	6.24E-06	6.47E-10	1.99E-06	2.27
FORMA	Formaldehyde	50-00-0	CH2O	30.03	1.75E-05	1.99E-09	3.37E-07	0.35
PEA	Phenethyl alcohol	60-12-8	C8H10O	122.16	7.21E-06	7.58E-10	2.56E-07	1.36
CAR	I-Carvone	2244-16-8	C10H14O	150.22	6.18E-06	6.76E-10	7.73E-05	2.71
AC	Acetophenone	98-86-2	C8H8O	120.15	7.18E-06	8.10E-10	1.04E-05	1.58
MB	Methyl Benzoate	93-58-3	C8H8O2	136.15	6.90E-06	7.70E-10	3.24E-05	2.12
HX	Hexanal	66-25-1	C6H12O	100.16	7.59E-06	8.10E-10	2.13E-04	1.78
PA	Phenyl Acetate	122-79-2	C8H8O2	136.15	6.90E-06	7.60E-10	6.48E-05	1.49
IAA	Isoamyl Acetate	123-92-2	C7H14O2	130.19	6.75E-06	7.12E-10	5.87E-04	2.25
AN	Anisole	100-66-3	C7H8O	108.14	7.67E-06	8.64E-10	4.84E-04	2.11
LM	D-Limonene	5989-27-5	C10H16	136.24	6.32E-06	6.84E-10	3.19E-02	4.57
VCH	Vinyl Cyclohexane	695-12-5	C8H14	110.2	7.13E-06	7.75E-10	1.76E-01	3.95
HP	Heptane	142-82-5	C7H16	100.21	7.23E-06	7.54E-10	2.00E+00	4.66
